# Spectrum Analysis of Inherited Metabolic Disorders for Expanded Newborn Screening in a Central Chinese Population

**DOI:** 10.3389/fgene.2021.763222

**Published:** 2022-01-12

**Authors:** Xia Li, Jun He, Ling He, Yudong Zeng, Xuzhen Huang, Yechao Luo, Yujiao Li

**Affiliations:** ^1^ Neonatal Disease Screening Center, Changsha Hospital for Maternal and Child Health Care, Changsha, China; ^2^ Technical Support Center, Zhejiang Biosan Biochemical Technologies Co., Ltd, Hangzhou, China

**Keywords:** inherited metabolic disorders, newborn screening, incidence rate, tandem mass spectrometry, mutation

## Abstract

Neonatal inherited metabolic disorders (IMDs) are closely associated with early neonatal death and abnormal growth and development. Increasing attention has been paid to IMDs because of their high incidence and diversity. However, there are no reports about the incidence of IMDs in Changsha, China. Therefore, we retrospectively analyzed the screening results of neonates to evaluate the characteristics of IMDs in the area. From January 2016 to December 2020, 300,849 neonates were enrolled for expanded newborn screening by tandem mass spectrometry in the Neonatal Disease Screening Center of the Changsha Hospital for Maternal & Child Health Care. Newborns with mild initial results were recalled for repeated tests; if the second test was still positive, the patient was referred for confirmatory tests. A total of 71 confirmed cases were identified in our study, with an incidence rate of 1:4,237. There were 28 cases of amino acid metabolic disorders, representing 39.44% of the IMDs diagnosed, with an incidence rate of 1:10,745. Twelve newborns were diagnosed with organic acid metabolic disorders, accounting for 16.66% of IMDs, with an incidence rate of 1:25,071. There were 31 cases of fatty acid oxidation disorders, representing 43.05% of IMDs, with an incidence rate of 1:9,705. Overall, 14 types of IMDs were found in Changsha. The most common disorders in the region were primary carnitine deficiency, hyperphenylalaninemia and short-chain acyl-CoA dehydrogenase deficiency. Their incidence rate is respectively 1:13,675, 1:16,714 and 1:42,978. The mutations in *PAH*, *SLC22A5*, and *ACADS* are the leading causes of IMDs in this area. This study demonstrates the importance of utilizing MS/MS in IMD screening for early diagnosis and treatment. This strategy may be used for prenatal genetic counseling to avoid irreversible growth and intellectual development disorders in children.

## Introduction

Inherited metabolic disorders (IMDs), also called inborn errors of metabolism, are a type of disease associated with abnormal accumulation of metabolites and deficiency of essential substances caused by metabolic pathway defects (Ferreira and van Karnebeek, 2019). To date, more than 3,000 types of IMDs have been identified (Mak et al., 2013), most of which have no clinical presentation at the early onset, which makes them difficult for clinicians to diagnose. Once abnormalities occur, they lead to intellectual disability, developmental delay, and even death. Therefore, early screening, diagnosis, and treatment of neonatal IMDs are of great importance for the prevention of birth defects (Martínez-Morillo et al., 2016). In 1990, tandem mass spectrometry (MS/MS) was expanded to the field of neonatal disease screening for the first time. Qualitative and quantitative analyses are performed by detecting the mass-to-charge ratio of substances in samples for screening. Dozens of metabolites can be analyzed simultaneously in 2 min, and more than 40 genetic metabolic diseases can be detected, including metabolic disorders of amino acids, organic acids, and fatty acids. At present, the use of MS/MS in screening of IMDs has been widely carried out in developed countries and regions, and the coverage rate of screening has also increased annually (Wilcken and Wiley, 2008). However, the incidence of IMDs detected by MS/MS varies greatly among different countries and regions. A pilot study of over seven million newborns in China between 2016 and 2017 revealed that the incidence of IMDs detected by MS/MS was 38.69 per 100,000 births (1:2,585) (Deng et al., 2021). However, data from the present study differed from what was reported by Deng et al. The disease spectrum and genetic background of neonates in the Changsha area that a city with a population of more than ten million, were elucidated for the first time in this study. The Neonatal Disease Screening Center of Changsha Hospital for Maternal & Child Health Care launched the screening of neonatal IMDs by MS/MS in 2014. It is the first institution in Hunan Province to carry out the screening of IMDs using MS/MS, and currently performs the largest amount of screening in the Hunan Province. The findings of this study are of great significance as a reference for the whole Hunan region.

## Materials and Methods

### Subjects

This study included 300,849 newborns born in six districts and two counties in Changsha from January 2016 to December 2020. Informed consent was obtained from the parents for the sample collection and testing.

### Methods

#### Sample Collection

After 72 h from birth, all newborns were fully breast-fed, and four drops of heel blood were collected by acupuncture and transferred to special blood collection cards (Schleicher and Schuell 903 filter paper) issued by our center. There were at least four blood spots on the filter paper, and the diameter was not less than 8 mm. The blood drops were allowed to penetrate naturally, and dry blood spots on the front and back sides of the filter paper were verified to be consistent and free from contamination. After the blood spots were naturally dried, they were placed in a sealed bag and stored in a refrigerator at 2–8°C. The samples were collected by logistics personnel within five weekdays and delivered to our central laboratory for testing.

#### Newborn Screening

**FIGURE 1:**
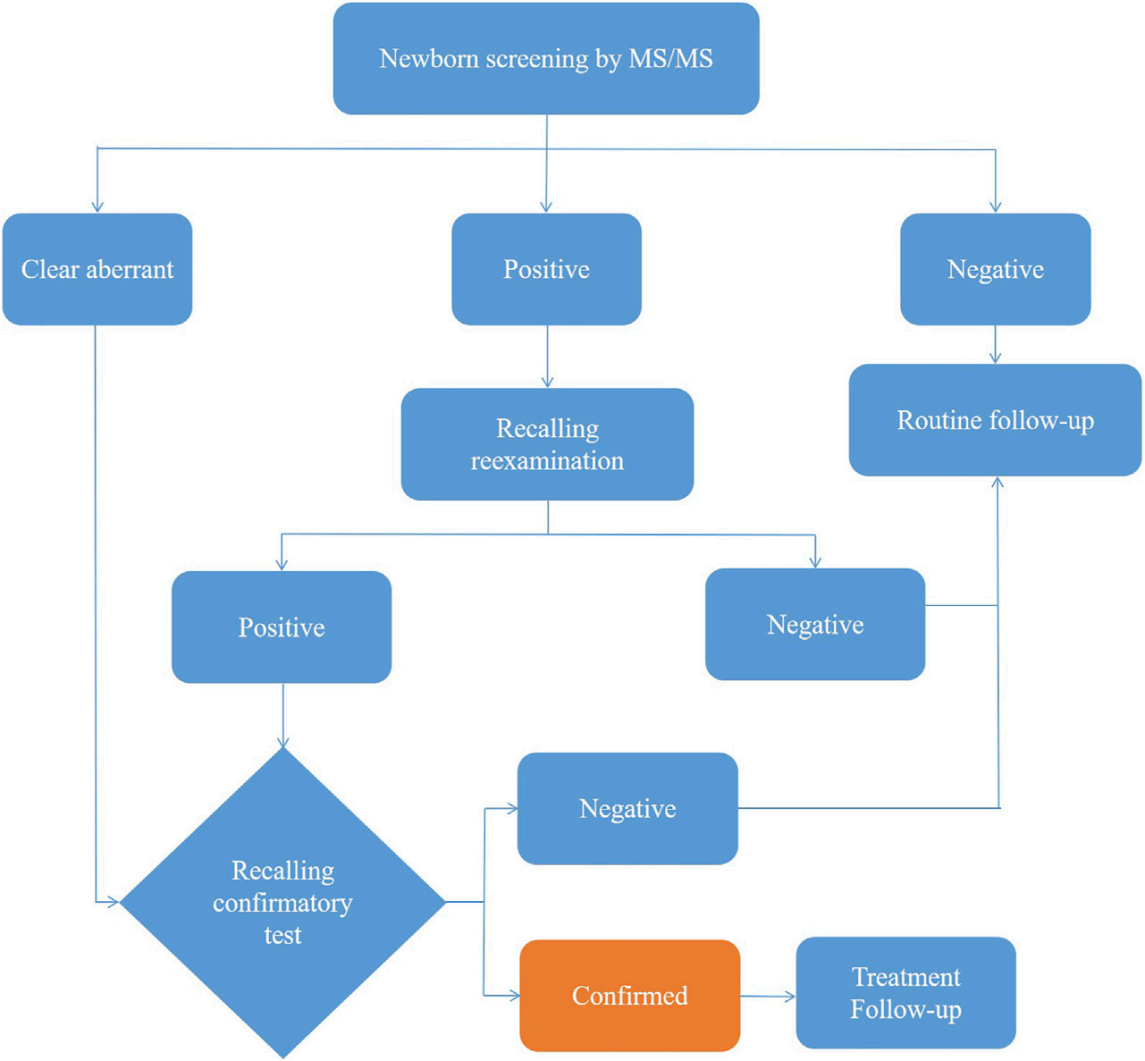
The flow diagram of newborn screening.

After extraction with an organic solvent containing an internal amino acid standard and acylcarnitine, samples were analyzed using a Waters TQD MS/MS screening system. The detection reagent was a NeoBase™ non-derivatized MS/MS Kit (tandem mass spectrometry, PerkinElmer, Finland), that has internal quality controls. After passing the internal quality control, data analysis and report distribution were carried out. There were 74 screening indicators (Table 1), including 11 amino acids, one ketone, and 31 acylcarnitines, and their ratios. The results were validated by regular external quality assessment by the National Center for Clinical Laboratories twice every year. The reference range of the laboratory was established according to the detection results by MS/MS, and metabolic analysis was carried out to determine whether the metabolism was normal or abnormal. Based on the metabolic characteristics, negative or positive screening results were obtained, and IMDs were diagnosed. The neonates who screened positive (suspected positive) for the first time were called and reexamined, if the second test was still positive, the patient was referred for confirmatory tests. Moreover, newborns with clear aberrant initial screening results were immediately referred for confirmatory tests. Then gas chromatography-mass spectrometry of urine and other auxiliary examinations were carried out. Suspected patients were further diagnosed by gene mutation detection. The flow program was shown in Figure 1. Case management, special diet or drug treatment, regular monitoring, and long-term follow-up were established for diagnosed children.

**TABLE 1:** The 74 indicators of newborn screening.

No	Screening indicators	Abbreviations	Cut-off values (μmol/L)	No	Ratios	Cut-off values
1	Free carnitine	C0	9–50	44	ARG/PHE	0.02–0.7
2	Acetylcarnitine	C2	3–48	46	MET/PHE	0.1–0.6
3	Propionylcarnitine	C3	0.35–4.0	47	MET/CIT	0.51–4
4	Malonylcarnitine+ 3-hydroxy butyrylcarnitine	C3DC + C4OH	0.03–0.4	48	ORN/CIT	2.5–22
5	Butyrylcarnitine	C4	0.1–0.5	49	ARG/ORN	0.01–0.4
6	Methylmalonylcarnitine+ 3-hydroxy isovalerylcarnitine	C4DC + C5OH	0.07–0.4	50	ALA/CIT	9.5–65
7	Isovalerylcarnitine	C5	0.03–0.3	51	CIT/PHE	0.1–0.7
8	Tiglylcarnitine	C5:1	0–0.03	52	PHE/TYR	0.2–1.4
9	Glutarylcarnitine+3-hydroxy hexanoylcarnitine	C5DC + C6OH	0.03–0.25	53	SA/PHE	0–0.04
10	Hexanoylcarnitine	C6	0.01–0.11	54	TYR/PHE	0.6–7
11	Adipylcarnitine	C6DC	0.03–0.27	55	(LEU + ILE + PRO-OH)/PHE	1.15–5.2
12	Octanoylcarnitine	C8	0.02–0.2	56	(LEU + ILE + PRO-OH)/TYR	0.5–4.2
13	Octenoylcarnitine	C8:1	0.03–0.45	57	C0/(C16 + C18)	1.8–30
14	Decenoylcarnitine	C10:1	0.02–0.17	58	C3/C0	0.01–0.2
15	Decanoylcarnitine	C10	0.02–0.3	59	C3/C2	0.03–0.2
16	Decadienoylcarnitine	C10:2	0–0.15	60	C3/MET	0.02–0.3
17	Dodecanoylcarnitine	C12	0.02–0.35	61	C4/C2	0–0.04
18	Dodecenoylcarnitine	C12:1	0.01–0.37	62	C4/C3	0.04–0.45
19	Myristoylcarnitine	C14	0.04–0.45	63	C5/C0	0–0.02
20	Myristoleylcarnitine	C14:1	0.02–0.35	64	C8/C2	0–0.01
21	Tetradecadienoylcarnitine	C14:2	0–0.05	65	C8/C10	0.3–1.5
22	3-hydroxy myristoylcarnitine	C14OH	0–0.05	66	C14:1/C2	0–0.02
23	Palmitoylcarnitine	C16	0.5–6.86	67	C14:1/C16	0.01–0.1
24	Hexadecenoylcarnitine	C16:1	0.02–0.5	68	C16OH/C16	0–0.02
25	3-hydroxy palmitoylcarnitine	C16OH	0–0.06	69	(C16 + C18:1)/C2	0.12–0.55
26	3-hydroxy palmitoleylcarnitine	C16:1OH	0.01–0.08	70	(C3DC + C4OH)/C10	0.35–4.33
27	Octadecanoylcarnitine	C18	0.24–2	71	(C5DC + C6OH)/(C3DC + C4OH)	0.3–2
28	Octadecenoylcarnitine	C18:1	0.38–3	72	(C5DC + C6OH)/(C4DC + C5OH)	0.15–1.6
29	Linoleylcarnitine	C18:2	0.05–0.6	73	(C0+C2+C3+C16 + C18:1 + C18)/CIT	1.3–12
30	3-hydroxy octadecanoylcarnitine	C18OH	0–0.03	74	(C4DC + C5OH)/C8	1–12
31	3-hydroxy octadecenoylcarnitine	C18:1OH	0–0.06	-		
32	Alanine	ALA	125–650			
33	Arginine	ARG	1–45			
34	Citrulline	CIT	5.5–26			
35	Glycine	GLY	190–1,000			
36	Leucine + isoleucine + hydroxyproline	LEU + ILE + PRO-OH	50–260			
37	Methionine	MET	6.5–40			
38	Ornithine	ORN	30–250			
39	Phenylalanine	PHE	23–100			
40	Proline	PRO	75–420			
41	Succinylacetone	SA	0–1.6			
42	Tyrosine	TYR	30–250			
43	Valine	VAL	40–230			

#### Positive Rules in Newborn Screening

41 kinds of IMDs were included in our screening panel. Each IMD had one or more screening indicators including metabolites and ratios, and their cut-off values. When the results met the positive rules of IMDs, they were considered as positive. All the positive rules of IMDs were indicated in Table 2.

**TABLE 2:** Positive rules in expanded newborn screening panel.

Disorders (OMIM code)	Positive rules 1	Positive rules 2	Positive rules 3	Positive rules 4	Positive rules 5
Phenylalanine hydroxylase deficiency (#261,600), Tetrahydrobiopterin deficiency (#233,910, #261,640, #612,716, #264,070, and #261,630)	PHE >100 μmol/L, PHE/TYR≥1.4	PHE >120 μmol/L	PHE/TYR≥2.4	-	-
Glutaric acidemia type Ⅰ (#231,670)	C5DC + C6OH > 0.25 μmol/L, (C5DC + C6OH)/(C3DC + C4OH) ≥ 2, (C5DC + C6OH)/(C4DC + C5OH) > 1.6	C5DC + C6OH > 0.3 μmol/L	-	-	-
Hyperornithinemia-hyperammonemia-homocitrullinuria syndrome (#238,970)	ORN >196 μmol/L, ORN/CIT >22	ORN >350 μmol/L	-	-	-
Citrullinemia type Ⅰ (#215,700), Citrin deficiency (Citrullinemia (#605,814 and #603,471), Argininosuccinic aciduira (#207,900)	CIT >26 μmol/L, ALA/CIT <9.5	CIT >65 μmol/L	-	-	-
Carbamoylphosphate synthetase I deficiency (#237,300), ornithine aminotransferase deficiency (#258,870), ornithine transcarbamylase deficiency (#311,250)	CIT <5.5 μmol/L, CIT/PHE <0.1	CIT <4.5 μmol/L	-	-	-
Tyrosinemia type I (#276,700)	SA > 1.1 μmol/L, SA/PHE≥0.04	SA > 1.8 μmol/L	-	-	-
Homocystinuria (#236,200), Hypermethioninemia (#250,850)	MET >40 μmol/L, MET/PHE >0.6	MET >65 μmol/L	-	-	-
Maple syrup urine disease (#248,600)	LEU + ILE + PRO-OH > 260 μmol/L, (LEU + ILE + PRO-OH)/PHE >5.2, VAL >230 μmol/L	LEU + ILE + PRO-OH > 400 μmol/L	-	-	-
Tyrosinemia type Ⅱ (276,600), Tyrosinemia type Ⅲ (#276,710)	TYR >250 μmol/L, (LEU + ILE + PRO-OH)/TYR <0.5, PHE/TYR <0.2	TYR >400 μmol/L	-	-	-
Argininemia (#207,800)	ARG/PHE >0.7, ARG/ORN>0.4, ARG>45	ARG >65 μmol/L	-	-	-
Methylmalonic acidemia (#251,000, #277,400, #277,410, #251,100, #251,110, #277,380, #309,541, #613, 646, #614, 265 and #614, 857), propionic acidemia (#606,054)	C3 > 4.5 μmol/L, C3/C2 > 0.2	C3 > 6.5 μmol/L	C3/C0 > 0.3	C3/C2 > 0.29	C3/MET >0.4
Isovaleric acidemia (#243,500), 2-methylbutryl CoA dehydrogenase deficiency (#610,006)	C5 > 0.3 μmol/L, C5/C0≥0.02	C5>0.8 μmol/L	-	-	-
Holocarboxylase synthetase deficiency (#253, 270), 3-methylglutaconyl CoA hydratase deficiency (#250,950), 3-methylcrotonyl CoA carboxylase deficiency (#210,200 and #210,210), 3-hydroxy-3-methylglutaryl-CoA lyase deficiency (#246,450)	C4DC + C5OH > 0.4 μmol/L, (C4DC + C5OH)/C0≥0.03	C4DC + C5OH > 0.5 μmol/L	-	-	-
Multiple acyl-CoA dehydrogenase deficiency (#231,680)	C5 > 0.3 μmol/L, C4 > 0.5 μmol/L	-	-	-	-
Beta-ketothiolase deficiency (#203,750)	C5:1≥0.01 μmol/L, C4DC + C5OH > 0.4 μmol/L	-	-	-	-
Malonic acidemia (#248,360)	C3DC + C4OH > 0.4 μmol/L, (C3DC + C4OH)/C10 > 4.33	C3DC + C4OH > 0.8 μmol/L	-	-	-
Medium chain acyl CoA dehydrogenase deficiency (#201,450)	C6 > 0.11 μmol/L, C8 > 0.2 μmol/L, C8/C2≥0.01, (C4DC + C5OH)/C8≤1	C8 > 0.3 μmol/L	-	-	-
Very long chain acyl CoA dehydrogenase deficiency (#201,475)	C14:1 > 0.35 μmol/L, C14:1/C16 > 0.1, C14:1/C2≥0.02	C14:1 > 0.5 μmol/L	-	-	-
Long chain 3-hydroxy acyl-CoA dehydrogenase deficiency (#609,016), trifunctional protein deficiency (#609,015)	C16OH > 0.06 μmol/L, C16OH/C16≥0.02, C18:1OH > 0.04 μmol/L, C18OH > 0.03 μmol/L	-	-	-	-
Primary carnitine deficiency (#212,140)	C0 < 8.5 μmol/L	C0 < 9.0 μmol/L, (C0+C2+C3+C16 + C18:1 + C18)/CIT≤1.3	-	-	-
Carnitine palmitoyltransferase I deficiency (#255,120)	C0/(C16 + C18) > 30, C0 > 50 μmol/L, (C16 + C18:1)/C2 < 0.12	C0 > 100 μmol/L	-	-	-
Carnitine palmitoyltransferase Ⅱ deficiency (#255,110, #608,836, #600,649), Carnitine/acylcarnitine translocase deficiency (#212,138)	(C16 + C18:1)/C2 > 0.55, C16 > 6.86 μmol/L, C0/(C16 + C18) ≤ 1.8	-	-	-	-
Short chain acyl CoA dehydrogenase deficiency (#201,470)	C4 > 0.5 μmol/L,C4/C2 > 0.04	C4 > 0.8 μmol/L	-	-	-
Hyperprolinuria (#239,500)	PRO >340 μmol/L	-	-	-	-
Nonketotic hyperglycinemia (#617, 301, #605,899)	GLY >1,000 μmol/L	-	-	-	-
Isobutyryl-CoA dehydrogenase deficiency (#611,283), ethylmalonic encephalopathy (#602,473)	C4/C3 > 0.45, C4/C2 > 0.04	C4 > 0.8 μmol/L	-	-	-

#### Detection of Organic Acids in Urine

Urine samples were collected from infants or prepared in urine filter paper. After urea removal, protein precipitation, oximation, and derivatization, the samples were analyzed by Agilent gas chromatography-mass spectrometry (6890-5977B) and quantified by the internal standard method.

#### Analysis of Urinary Pterin

Neonatal urine is collected in a light proof container, ascorbic acid (10 mg/ml) should be added immediately. After mixing, it should be kept away from light at −20°C or transferred to 5 × 5 cm special filter paper. The urinary filter paper with 4.7 mm in diameter were punched and added with 1 ml purified water, filtrating with chromatography column. The supernatant was centrifugated after adding MnO_2_. And then it was analysed by using Shimadzu high performance liquid chromatography (LC-20ADXR).

#### Determinating the Activity of Dihydropteridine Reductase

Two drops of heel blood were collected from the suspected positive neonate by acupuncture and transferred to special blood collection cards (Schleicher and Schuell 903 filter paper). 5 mm diameter blood spot was punched and put into the centrifugal tube. Afrer adding potassium chloride, shaking and centrifugating, then determinating by Shimadzu ultraviolet spectrophotometer (UV2600).

#### Genetic Analysis

Genomic DNA was extracted from dry blood spots or peripheral blood of suspected positive patients using the Qiagen blood DNA Mini Kit. Eighty-six capture probes for *PAH*(OMIM# 612,349), *PTS* (OMIM# 612,719), *ACADS* (OMIM# 606,885), *SLC22A5* (OMIM# 603,377) and other genes related to IMDs were customized by Agilent, and the target region sequences were enriched by multiple probe hybridization (Supplementary Table S1). The capture products were purified using Agencourt AMPure XP magnetic beads (Beckman Coulter). Genomic DNA was sheared to an approximate mean fragment length of 200 base pairs using Covaris LE220. Sheared DNA was used for library preparation of the targeted regions by multiplex polymerase chain reaction. DNA samples of the probands were quantified using a Qubit dsDNA HS Assay Kit and then used for next-generation sequencing. All variants identified were further validated by Sanger sequencing using an ABI 3500XL.

#### Follow-Up

Patients’ height, weight, and head circumference were followed up every 3 months up till 1 year of age, every 6 months from one to 3 years of age, and every 6 months to 1 year after 3 years of age. The developmental quotient (DQ) or intelligence quotient (IQ) was evaluated every one to 3 years after birth, and the following methods of intelligence measurement were used for each age: the Gesell Developmental Scale for children < three years old, the Wechsler Preschool Scale of Intelligence (WPPSI) for four to six years old, and the Wechsler Intelligence Scale for School-Age Children (WISC-R) for > six years old. DQ or IQ > 70 was considered normal. Individuals were closely monitored during treatment for adverse drug reactions and adaptation to feeding practices.

#### Statistical Analysis

All data were analyzed by SPSS 21.0 statistical software. The positive rate was calculated using the percentage method.

## Results

### Screening Situation

**FIGURE 2:**
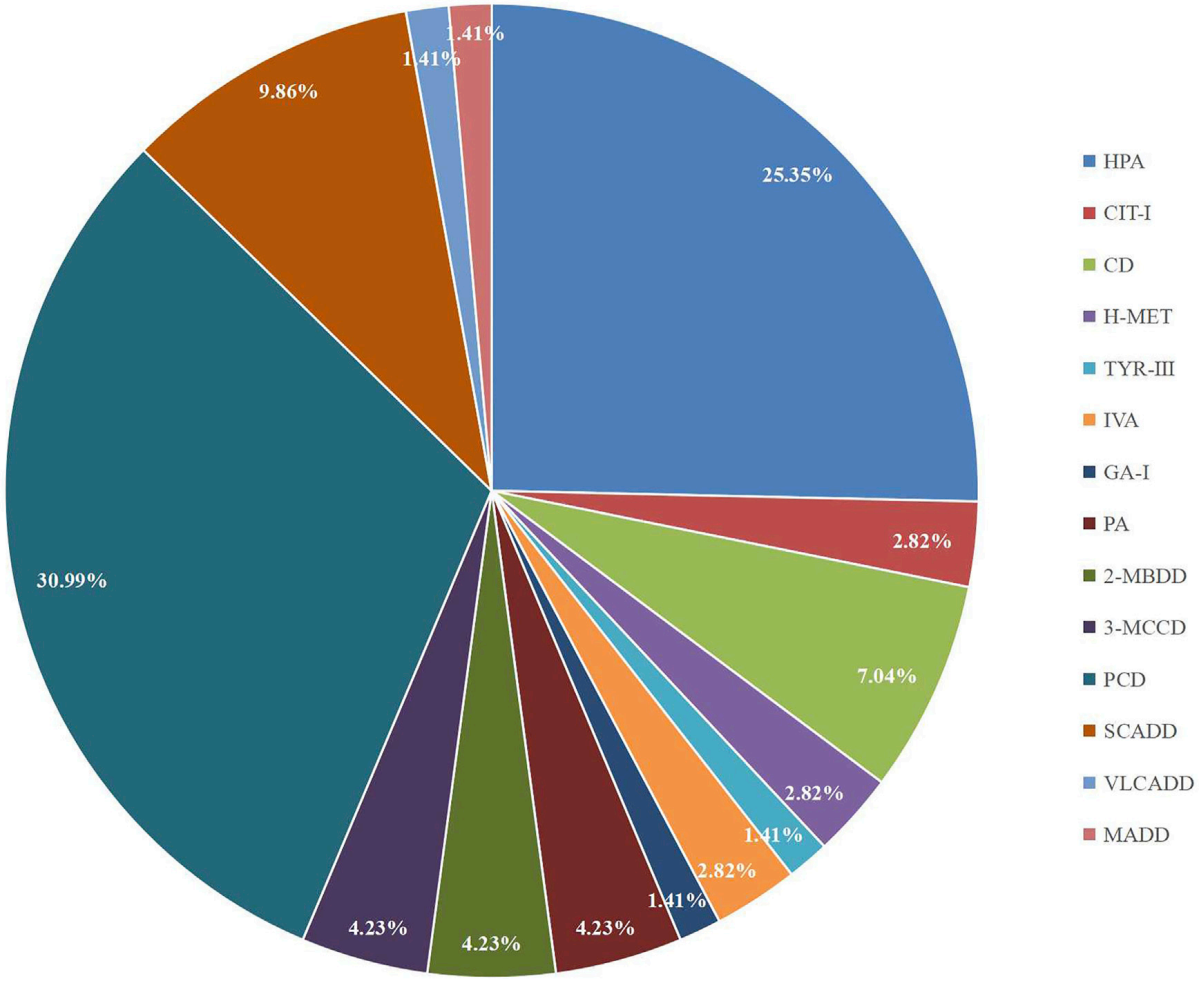
The proportion of different types of IMDs in Changsha.

A total of 300,849 newborns (178,265 males and 122,584 females) were screened; 4,923 newborns were suspected to be positive in the first screening, 4,315 newborns were successfully recalled for retesting (87.65%), and 71 newborns were confirmed to have IMDs, where 14 types of IMDs were found. (Figure 2 and Table 3).

**TABLE 3:** Statistical analysis of neonatal screening for IMDs in Changsha from 2016 to 2020.

Year	Number of screenings	Suspected positive number	Actual recall number	Recall rate (%)	Confirmed number	Frequency
2016	39,602	435	348	80.00	8	1: 4,950
2017	66,399	839	671	79.98	13	1: 5,107
2018	66,037	657	598	91.02	16	1: 4,127
2019	69,372	1,444	1,302	90.16	14	1: 4,955
2020	59,439	1,548	1,396	90.18	20	1: 2,971
Total	300,849	4,923	4,315	87.65	71	1: 4,237

### Laboratory Test and Gene Analysis of Children With Positive Diagnosis

Among the 300,849 newborns, 71 were diagnosed with neonatal metabolic diseases (44 males and 27 females), with an overall incidence rate of 1:4,237 (Table 4). There were 28 cases of amino acid metabolic disorders (AAMDs) diagnosed, accounting for 39.44% of IMDs, with an incidence rate of 1:10,745 (Table 4). Twelve newborns were diagnosed with organic acid metabolic disorders (OAMDs), which represented 16.66% of the proportion of IMDs, with an incidence rate of 1:25,071 (Table 4). We found 31 cases of fatty acid oxidation disorders (FAODs), which accounted for 43.05% (Figure 2) of IMDs, with an incidence rate of 1:9,705 (Table 4). Primary carnitine deficiency (PCD, OMIM# 212,140) (1:13,675, 30.99%) (Figure 2 and Table 4), hyperphenylalaninemia (HPA, OMIM# 261,600, 233,910, 261,640, 612,716, 264,070, and 261,630) (1:16,714, 25.35%) and short chain acyl CoA dehydrogenase deficiency (SCADD, OMIM# 201,470) (1:42,978, 9.86%) (Figure 2 and Table 4) were the most common disorders in the region. Tyrosinemia type Ⅲ (TYR-Ⅲ, OMIM# 276,700, 276,600, 276,710), glutaric acidemia type Ⅰ (GA-Ⅰ, OMIM# 231,670), very long chain acyl CoA dehydrogenase deficiency (VLCADD, OMIM# 609,016) and multiple acyl CoA dehydrogenase deficiency (MADD, OMIM# 231,680) were the least common disorders, with a same incidence rate of 1:300,849. Among the patients, one newborn with propionic acidemia (PA, OMIM# 606,054) died prematurely, and another one with PA and six newborns with HPA only obtained clinical diagnosis results because their parents chose other medical institutions for diagnosis and treatment; therefore, they did not undergo complete laboratory testing and genetic diagnosis (Table 8). All the detection results of the cases with IMDs were shown in Table 5–8.

**TABLE 4:** Screening results of 300,849 cases of IMDs by MS/MS.

Disorders (OMIM codes)	Confirmed cases	Frequency
**Amino acid metabolic disorders**	**28**	**1:10,745**
Phenylalanine hydroxylase deficiency (#261,600)	16	1:18,803
Tetrahydrobiopterin deficiency (#233,910, #261,640, #612,716, #264,070, and #261,630)	2	1:150,425
Citrullinemia type Ⅰ (#215,700)	2	1:150,425
Citrin deficiency (#605,814 and #603,471)	5	1:60,170
Hypermethioninemia (#250,850)	2	1:150,425
Tyrosinemia type Ⅲ (#276,710)	1	1:300,849
**Organic acid metabolic disorders**	**12**	**1:25,071**
Isovaleric acidemia (#243,500)	2	1:150,425
Glutaric acidemia type Ⅰ (#231,670)	1	1:300,849
Propionic acidemia (#606,054)	3	1:100,283
2-methylbutryl CoA dehydrogenase deficiency (#611,283)	3	1:100,283
3-methylcrotonyl CoA carboxylase deficiency #(210,200 and #210,210)	3	1:100,283
**Fatty acid oxidation disorders**	**31**	**1:9,705**
Primary carnitine deficiency (#212,140)	22	1:13,675
Short chain acyl CoA dehydrogenase deficiency (#201,470)	7	1:42,978
Very long chain acyl CoA dehydrogenase deficiency (#609,016)	1	1:300,849
Multiple acyl CoA dehydrogenase deficiency (#231,680)	1	1:300,849
**Total**	**71**	**1:4,237**

All the inherited metabolic disorders are classified as amino acid metabolic disorders, organic acid metabolic disorders, and fatty acid oxidation disorders, we bolded them only to distinguish from the single disorder.

**TABLE 5:** Biochemical and genetic data of positive cases with AAMDs.

No	Gender	Confirmed age (day)	Abnormal level (μmol/L)	References range (μmol/L)	Affected gene	Allele 1		Allele 2	
						Nucleotide variant	Amino acid variant	Nucleotide variant	Amino acid variant
1	M	47	Phe: 444.79	23–100	*PTS* (*612,719)	c.84–291A > G	-	c.286G > A	p.D96N
2	M	41	Phe: 245.11	23–100		c.4A > C	p.S2R	c.259C > T	p.P87S
3	M	30	Phe: 105.43	23–100	*PAH* (*612,349)	c.611A > G	p.Y204C	c.158G > A	p.R53H
4	F	47	Phe: 132.91	23–100		c.728G > A	p.R243Q	c.158G > A	p.R53H
5	M	33	Phe: 218.86	23–100		c.721C > T	p.R241C	-	-
6	M	50	Phe: 745.62	23–100		c.208_210delTCT	p.S70del	c.353–6T > C	-
7	F	36	Phe: 562.35	23–100		c.1068C > A	p.Y356*	c.907del	p.S303Pfs*38
8	M	31	Phe: 502.76	23–100		c.611A > G	p.Y204C	c.728G > A	p.R243Q
9	F	30	Phe: 159.60	23–100		c.527G > A	p.R176Q	c.498C > G	p.Y166*
10	M	19	Phe: 135.63	23–100		c.1068C > A	p.Y356*	c.158G > A	p.R53H
11	F	34	Phe: 264.59	23–100		c.721C > T	p.R241C	c.284_286delTCA	p.I95del
12	F	27	Phe: 160.25	23–100		c.464G > A	p.R155H	c.331C > T	p.R111*
13	F	35	Met: 46.97	6.5–40	*MAT1A* (*610,550)	c.755T > C	p.I252T	-	
14	F	27	Met: 75.66	6.5–40		c.314A > T	p.N105I	c.386A > G	p.D129G
15	M	33	TYR: 447.22	30–250	*HPD* (*609,695)	c.893A > C	p.Q298P	c.217T > C	p.S73P
16	F	54	CIT: 350.67	5.5–26	*ASS1* (*603,470)	c.1087C > T	p.R363W	c.748C > T	p.L250F
17	M	77	CIT: 39.00	5.5–26		c.1087C > T	p.R363W	c.11A > G	p.K4R
18	F	21	CIT: 186.21	5.5–26	*SLC25A13* (*603,859)	c.1048G > A	p.D350N	IVS16ins3kb	p.A584Vfs*2
19	M	25	CIT: 72.41	5.5–26		IVS16ins3kb	p.A584Vfs*2	c.852_855delTATG	p.M285Pfs*2
20	M	50	CIT: 92.49	5.5–26		c.852_855delTATG	p.M285Pfs*2	-	
21	M	43	CIT: 40.68	5.5–26		c.852_855delTATG	p.M285Pfs*2	c.1638_1660dup23	p.A554Gfs*17
22	F	40	CIT: 25.83	5.5–26		c.852_855delTATG	p.M285Pfs*2	c.1750_1751ins3kb	-

M: male; F: female; -: no mutation.

**TABLE 6:** Biochemical and partial genetic data of children with OAMDs.

No	Gender	Confirmed age (day)	Abnormal level (μmol/L)	References range (μmol/L)	Affected gene	Allele 1		Allele 2	
						Nucleotide variant	Amino acid variant	Nucleotide variant	Amino acid variant
1	M	23	C4DC + C5OH: 0.83	0.07–0.4	*MCCC2* (*609,010)	c.730C > G	p.P244A	c.1144_1147inv	p.K382_K383delinsF*
2	M	30	C4DC + C5OH: 1.13	0.07–0.4	-	c.1103delG	p.G368Vfs*70	c.1550G > A	p.G517E
3	M	78	C4DC + C5OH: 3.5	0.07–0.4	-	c.1061C > T	p.T354l	c.1599T > A	p.D533E
4	M	31	C5: 0.43	0.03–0.3	*ACADSB* (*610,006)	c.1165A > G	p.M389V	-	-
5	M	41	C5: 0.48	0.03–0.3		c.1165A > G	p.M389V	-	-
6	M	41	C5: 0.83	0.03–0.3		c.1165A > G	p.M389V	-	-
7	M	33	C3: 4.28	0.35–4	*PCCA* (*232,000)	c.819+1G > A	-	c.1850T > C	p.L617P
8	F	16	C5DC + C6OH: 2.49	0.03–0.25	*GCDH* (*608,801)	c.532G > A	p.G178R	c.1244–2A > C	-
9	F	16	C5: 9.12	0.03–0.3	*IVD* (*607,036)	c.158G > C	p.Arg53Pro	c.349G > A	p.Glu117Lys
10	F	34	C5: 1.41	0.03–0.3	-	c.631A > G	p.T211A	c.865G > A	p.G289R

**TABLE 7:** Biochemical and genetic data of children with FAODs.

No	Gender	Confirmed age (day)	Abnormal level (μmol/L)	References range (μmol/L)	Affected gene	Allele 1		Allele 2		Allele 3	
						Nucleotide Variant	Amino acid Variant	Nucleotid Variante	Amino acid Variant	Nucleotide Variant	Amino acid Variant
1	M	35	C0: 5.15	9–50	*SLC22A5* (*603,377)	c.1400C > G	p.S467C	c.428C > T	p.P143L	-	--
2	M	17	C0: 4.36	9–50	-	c.51C > G	p.F17L	-		-	-
3	M	19	C0: 4.23	9–50	-	c.760C > T	p.R254X	c.1400C > G	p.S467C	-	-
4	F	23	C0: 2.79	9–50	-	c.497+1G > T	-	-		-	-
5	F	36	C0: 6.13	9–50	-	c.1400C > G	p.S467C	-		-	-
6	F	23	C0: 5.24	9–50	-	c.51C > G	p.F17L	c.621G > T	p.Q207H	-	-
7	F	40	C0: 5.11	9–50	-	c.431T > C	p.L144P	c.1195C > T	p.R399W	-	-
8	M	28	C0: 5.19	9–50	-	c.51C > G	p.F17L	c.338G > A	p.C113Y	-	-
9	M	59	C0: 5.28	9–50	-	c.1195C > T	p.R399W	c.1400C > G	p.S467C	-	-
10	M	33	C0: 7.54	9–50	-	c.1400C > G	p.S467C	c.1196G > A	p.R399Q	-	-
11	M	154	C0: 4.39	9–50	-	c.51C > G	p.F17L	c.470C > T	P.S157F	-	-
12	M	31	C0: 1.94	9–50	-	c.338G > A	p.C113Y	-		-	-
13	F	22	C0: 2.47	9–50	-	c.1108G > A	p.G370R	c.51C > G	p.F17L	-	-
14	F	38	C0: 2.97	9–50	-	c.760C > T	p.R254X	-		-	-
15	M	51	C0: 5.18	9–50	-	c.51C > G	p.F17L	c.782_799del	p.V261_P266del	-	-
16	F	38	C0: 4.94	9–50	-	c.760C > T	p.R254X	c.845G > A	p.R282Q	-	-
17	F	46	C0: 6.72	9–50	-	c.1400C > G	p.S467C	-		-	-
18	F	23	C0: 3.52	9–50	-	c.760C > T	p.R254X	c.1400C > G	p.S467C	-	-
19	M	37	C0: 4.88	9–50	-	c.760C > T	p.R254X	c.1400C > G	p.S467C	-	-
20	M	25	C0: 3.08	9–50	-	c.51C > G	p.F17L	c.653–8T > A	-	-	-
21	M	56	C0: 4.99	9–50	-	c.51C > G	p.F17L	-		-	-
22	F	56	C0: 3.25	9–50	-	c.760C > T	p.R254X	-		-	-
23	F	31	C12: 0.66; C14: 2.76; C18: 2.23	C12: 0.02–0.35; C14: 0.04–0.45; C18: 0.24–2	*ACADVL* (*609,575)	c.621_622+9del	-	c.622 + 14del	-	c.1531C > T	p.R511W
24	M	53	C5: 0.45 (C5DC + C6OH)/(C3DC + C4OH): 5.0	C5: 0.03–0.3; C5DC + C6OH)/(C3DC + C4OH:0.3–2	*ETFB* (*130,410)	c.340_342del	p.Lys114del	c.253C > T	p.Arg85Ter	c.82G > A	p.Gly28Ser
25	M	41	C4: 1.46	0.1–0.5	*ACADS* (*606,885)	c.172C > T	p.R58*	c.286G > A	p.G96S	-	-
26	M	26	C4: 1.20	0.1–0.5	-	c.220C > T	p.P74S	c.413del	p.N138Mfs*36	-	-
27	M	38	C4: 1.41	0.1–0.5	-	c.1031A > G	p.E344G	-		-	-
28	M	49	C4: 1.83	0.1–0.5	-	c.413delA	p.N138Mfs*36	c.758T > G	p.V253G	-	-
29	M	59	C4: 0.91	0.1–0.5	-	c.1130C > T	p.P377L	c.578C > T	p.S193L	-	-
30	M	30	C4: 1.75	0.1–0.5	-	c.795+1G > A	-	c.1031A > G	p.E344G	-	-
31	F	140	C4: 1.61	0.1–0.5	-	c.578 > T	p.S193L	c.1031A > G	p.E344G	-	-

**TABLE 8:** Eight cases without genetic diagnosis data.

No	Gender	Confirmed age (day)	Abnormal level (μmol/L)	Analysis of urinary pterin (mmol/mol Cre)	DHPR activity (nmol/min/mg Hb)	Determination of organic acid in urine	Diagnosis
1	M	40	Phe: 701.03↑	N: 1.43; B: 0.38↓; B%: 21.00	1.17	-	HPA
2	M	60	Phe: 563.53↑	N: 1.46; B: 0.58↓; B%: 28.43	2.46	-	HPA
3	F	75	Phe: 498.89↑	N: 4.26; B: 0.61↓; B%: 12.53↓	1.90	-	HPA
4	M	42	Phe: 148.76↑	N: 4.01↑; B: 0.57; B%: 14.21↓	1.63	-	HPA
5	M	56	Phe: 269.3↑	N: 4.11↑; B: 0.47; B%: 10.26↓	2.00	-	HPA
6	F	50	Phe: 172.9↑	N: 1.51; B: 0.60; B%: 28.44	1.70	-	HPA
7	M	20	C3: 10.45↑	-	-	3-hydroxypropionic acid↑, alanyl glycine↑, methyl citric acid↑, methyl Crotonyl glycine↑	PA
8	M	-	C3: 17.89↑	-	-	oxalic acid↑, 3-hydroxypropionic acid↑, 3-hydroxybutyric acid↑, 3-hydroxyisovaleric acid↑, alanyl glycine, 5-oxo-proline↑, methyl citric acid↑, methyl Crotonyl glycine↑	PA

“↑” or “↓” indicates that the detected levels of metabolic markers are above or below the reference range.

N: neopterin, B: biopterin, B%: B/(N + B)×l00%.

DHPR: dihydropteridine reductase.

### Follow-Up and Treatment

The follow-up situations of 71 confirmed cases were demonstrated in Table 9, two cases of PA came from the same family and were born at different times. One patient died due to multiple organ failure at birth, which was confirmed by the left samples. The latter case and the other six cases of HPA were not filed in our center because their parents chose another hospital for diagnosis and treatment. One case of citrin deficiency (CD, OMIM# 605814 and 603471) was found to be normal by primary screening with MS/MS, but 40 days after birth, the child was confirmed to have repeated jaundice. The patient’s condition improved after stopping breast milk and treatment with phenobarbital and ursodeoxycholic acid. The child was treated with a lactose-free formula as in the other four cases of CD. Their liver and kidney functions were checked regularly for half a year, and the results were normal. There were 22 cases of PCD confirmed by sequencing technology, and they were treated with l-carnitine. During the treatment, the blood was reexamined every 1–3 months to observe the levels of free carnitine and other acylcarnitine in order to adjust the treatment dosage. No clinical symptoms were observed during follow-up. Twelve children with HPA were treated with a special diet and drugs under the guidance of newborn screening specialist doctors. Their growth and intelligence were similar to those of normal children of the same age. Seven newborns with SCADD were given dietary guidance to avoid starvation, supplemented with carnitine, and examined regularly. One patient with VLCADD was also given dietary guidance to avoid fasting, and was prescribed a high carbohydrate, low fat diet, especially to limit the intake of long-chain fatty acids. One case of PA, one case of GA-Ⅰ, and two cases of isovaleric acidemia (IVA, OMIM# 243,500) were recommended a low-protein diet, special formulas, and oral l-carnitine for treatment. They were ordered for reexamination once every 3 months. Three cases of 2-methylbutyryl-CoA dehydrogenase deficiency (2-MBDD, OMIM# 611,283) and three cases of 3-methylcrotonyl-CoA carboxylase deficiency (3-MCCD, OMIM# 210,200 and 210,210) were not given special treatment, and their condition was normal during the follow-up. If the carnitine level was very low, l-carnitine was supplemented, and it was suggested to limit isoleucine and low protein in the long-term diet and to avoid long-term starvation. One patient with MADD was in a normal condition after birth. He was required to regularly follow up and monitor the indicators. When clinical symptoms appear, he should actively receive symptomatic support treatment and l-carnitine and vitamin B supplements. One patient with TYR-III was given a low phenylalanine and low tyrosine diet. Two patients with citrullinemia type I (CIT-I, OMIM# 215,700) had a long-term restriction on protein intake, used special milk powder, and regularly monitored blood ammonia values to avoid infection and prevent the occurrence of high blood ammonia. Two patients with hypermethioninemia (H-MET, OMIM# 250,850) did not show any clinical symptoms at birth. They were ordered to regularly monitor the concentration of methionine and to control the diet. Among the 71 confirmed cases, one patient died prematurely, 63 patients had normal physical and mental development, and there were no cases of acute metabolic disorder. The follow-up of seven children diagnosed and treated in another hospital showed a stable condition with no abnormality.

**TABLE 9:** The follow-up situations of IMDs.

Disorders (OMIM code)	Follow-up		
	Normal development and growth (case)	Developmental delay and intellectual disability (case)	Death (case)
Phenylalanine hydroxylase deficiency (#261,600)	16	0	0
Tetrahydrobiopterin deficiency (#233,910, #261,640, #612,716, #264,070, and #261,630)	2	0	0
Citrin deficiency (#605814 and #603,471)	5	0	0
Citrullinemia type Ⅰ (#215,700)	2	0	0
Hypermethioninemia (#250,850)	2	0	0
Tyrosinemia type Ⅲ (#276,700, #276,600, #276,710)	1	0	0
Isovaleric acidemia (#243,500)	2	0	0
Propionic acidemia (#606,054)	2	0	1
Glutaric acidemia type Ⅰ (#231,670)	1	0	0
2-methylbutryl CoA dehydrogenase deficiency (#611,283)	3	0	0
3-methylcrotonyl CoA carboxylase deficiency (#210,200 and #210,210)	3	0	0
Primary carnitine deficiency (#212,140)	22	0	0
Short chain acyl CoA dehydrogenase deficiency (#201,470)	7	0	0
Very long chain acyl CoA dehydrogenase deficiency (#609,016)	1	0	0
Multiple acyl CoA dehydrogenase deficiency (#231,680)	1	0	0
**Total**	70	0	1

## Discussion

The incidence rate of IMDs in Changsha varies greatly across countries. The incidence rate of IMDs in California, United States, is 1:3,367 (Frazier et al., 2006), 1:2,960 in Spain (la Marca et al., 2008), 1:2,396 in Portugal (Vilarinho et al., 2010), and 1:2,855 in Australia (Kasper et al., 2010). In Asian countries, the incidence rate of IMDs in Korea is 1:13,205; 1:2,916 in Malaysia; 1:3,159 in Singapore; and 1:8,557 in Japan (Yoon et al., 2005; Lim et al., 2014; Yunus et al., 2016; Shibata et al., 2018). The incidence rates of IMDs vary in different regions of China. For example, the incidence rate of IMDs in Jining is 1:1,178 (Guo et al., 2018), which is higher than that in Changsha, but only seven kinds of diseases were found. Yiming reported that the overall incidence rate in Quanzhou was 1:2,804 (Lin et al., 2019); however, a study showed that the incidence rate of IMDs in Zhejiang is 1:5,626 (Huang et al., 2011). In this study, we found that the overall incidence rate of IMDs detected by MS/MS in Changsha was 1:4,237, which is dissimilar to the incidence rates reported in Jining, Quanzhou, and Zhejiang.

**FIGURE 3:**
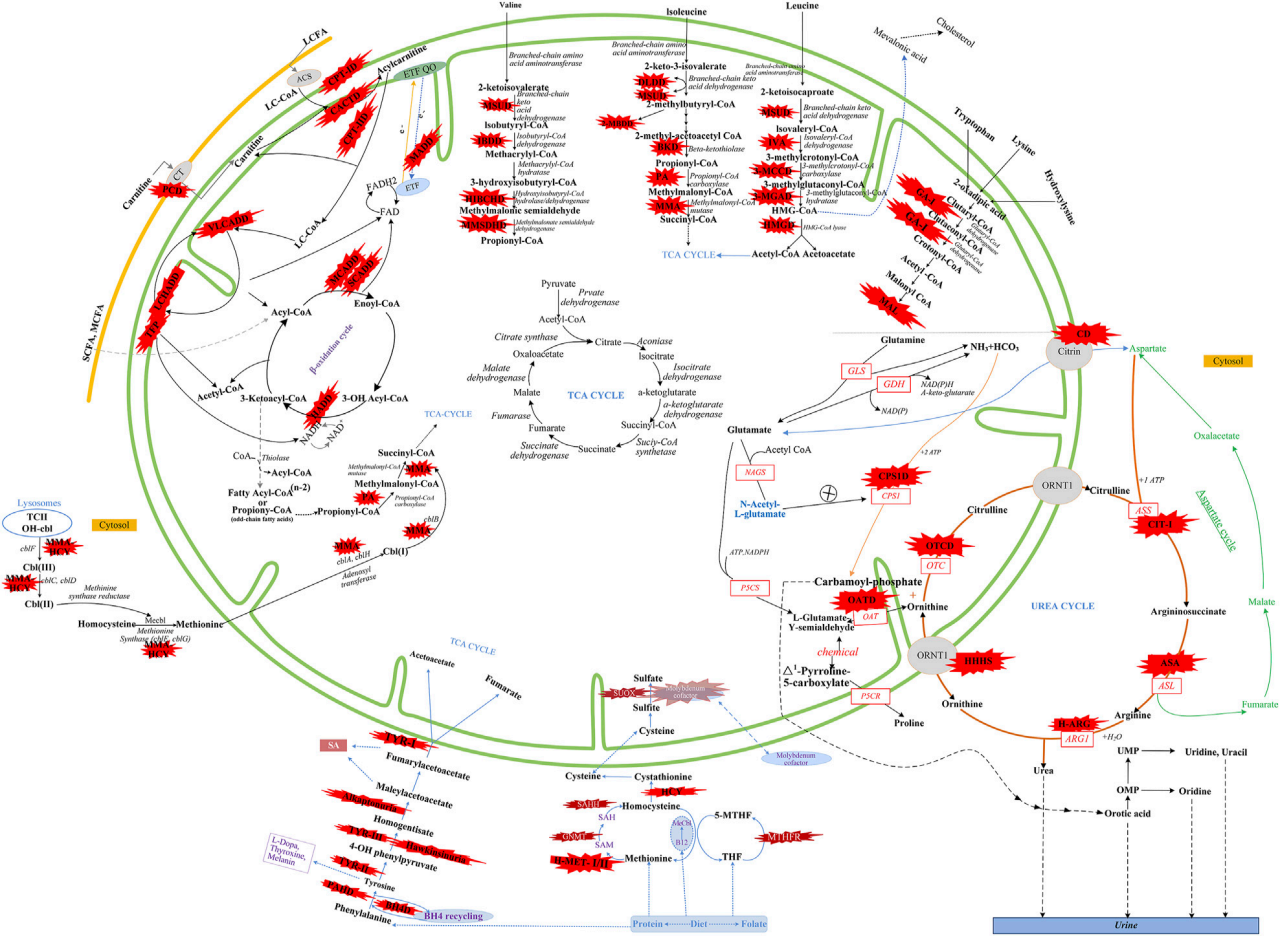
Overview of IMDs metabolism. The IMDs are shown in the red star shape. Green line represents mitochondrial membrane. Yellow line represents cell membrane. Adocbl, adenosylcobalamin; ACS, acyl-CoA synthase; B12, vitamin B12; C I, complex I of electron transfer chain; CT, carnitine transporter; ARG1, arginase 1; ASL, argininosuccinate lyase; argininosuccinate synthetase; ETF, electron transfer flavoprotein; FA, fatty acids; GDH, glutamate dehydrogenase; GLS, glutaminase; LC, Long Chain; MC, medium chain; MeCbl, methylcobalamin; MTHFR, methylene tetrahydrofolate reductase; NAD(P), nicotinamide adenine dinucleotide (phosphate); OAT, ornithine aminotransferase; OH-cbl, hydroxycobalamin; OMP, orotidine monophosphate; OTC, ornithine transcarbamylase; P5CR, pyrroline-5-carboxylate reductase; P5CS, Δ1-pyrroline-5-carboxylate synthetase; SAH, S-adenosylhomocysteine; SAHH, S-adenosylhomocysteine hydrolase; SAM, S-adenosylmethionine; SUOX, sulfite oxidase; TCⅡ, transcobalamin Ⅱ; TCA cycle, tricarboxylic acid cycle; THF, tetrahydrofolate; UMP, uridine monophosph. Modified from Aliu et al., 2018, Ramsay et al., 2018, Häberle et al., 2012, and Merritt et al., 2018.

There were five kinds of AAMDs in Changsha, with HPA and CD having the two highest incidence rates. HPA is a group of the most common AAMDs due to the deficiency of phenylalanine hydroxylase or tetrahydrobiopterin, resulting in elevated phenylalanine. The biochemical pathway of phenylalanine metabolism was illustrated in Figure 3. The incidence rate of HPA is reportedly between 1:2,600 and 1:143,000 (Mitchell et al., 2011). It has been previously reported that the incidence rate of HPA in China is 1:11,572 (Zhan et al., 2009), and the incidence rate of HPA is significantly high in the north. The incidence rate of HPA in Changsha is 1:16,714, higher than that in Zhejiang (1:20,445), and lower than that in Jining (1:4,391), a northern city in China (Guo et al., 2018), which is consistent with previous reports. c.158G > A is the most common hotspot mutation of *PAH* (OMIM* 612,349), which accounting for 12.5% of mutations in HPA (Table 10). 17 kinds of mutations were found and the total mutations associated with HPA that have accounted for 20.34% of all mutations in our patients with IMDs. Citrullinemia is divided into CIT-Ⅰ and citrullinemia type Ⅱ (CD), both of which are autosomal recessive disorders of the urea cycle. Citrullinemia type Ⅱ is due to mutations in the *SLC25A13* gene (OMIM* 603,859) (localized at 7q21.3), which encodes Citrin, causing impaired urea cycling and NADH transport and associated metabolic disturbances, The urea cycle and associated pathways were shown in Figure 3. The overall incidence rate of CD is 1:60,170 in Changsha, which is consistent with previous reports (Wang et al., 2019). According to a previous report, IVS16ins3kb, c.852_855delTATG are hotspot mutation sites in Chinese patients with CD (Lin et al., 2016). The results of this study are consistent with this report, and these two mutations accounted for 66.67% of the total mutations in *SLC25A13* (Table 10). In one case of CD, the concentration of citrulline at initial screening was normal, but CD was confirmed by genetic testing, suggesting that early detection is of great importance for diagnosis, but not all patients with CD had an increase in citrulline (Lin et al., 2020). Two cases of neonatal CIT-I and H-MET were found, with an incidence rate of 1:150,425. The incidence rate of TYR-Ⅲ was the lowest, with an incidence rate of 1:300,849.

**TABLE 10:** Mutations detected in patients with IMDs identified by expanded newborn screening.

Disorders (OMIM number)	Gene (OMIM number)	Mutation alleles number	Transcript	Nucleotide variant	Amino acid variant	Pathogenic	Relative frequency (%)	Accounting for total mutations (%)
Primary carnitine deficiency (#212,140)	*SLC22A5* (*603,377)	37	-	-	-	-	-	31.36
		9	NM_003,060.4	C.1400C > G	p.S467C	P	24.32	7.63
		8		C.51C > G	p.F17L	VUS	21.62	6.78
		6		C.760C > T	p.R254X	P	16.22	5.08
		2		C.1195C > T	p.R399W	P	5.41	1.69
		2		C.338G > A	p.C113Y	P	5.41	1.69
		1		C.1108G > A	p.G370R	VUS	2.70	0.85
		1		C.1196G > A	p.R399Q	P	2.70	0.85
		1		C.428C > T	p.P143L	LP	2.70	0.85
		1		C.431T > C	p.L144P	VUS	2.70	0.85
		1		C.470C > T	P.S157F	VUS	2.70	0.85
		1		C.497+1G > T	-	P	2.70	0.85
		1		C.621G > T	p.Q207H	LP	2.70	0.85
		1		C.653–8T > A		VUS	2.70	0.85
		1		C.782_799del	p.V261_P266del	VUS	2.70	0.85
		1		C.845G > A	p.R282Q	P	2.70	0.85
Hyperphenylalaninemia		24	-	-	-	-	-	20.34
Phenylalanine hydroxylase deficiency (#261,600)	*PAH* (*612,349)	20	-	-	-	-	83.33	16.95
		3	NM_000,277.3	C.158G > A	p.R53H	VUS	12.50	2.54
		2		C.1068C > A	p.Y356*	P	8.33	1.69
		2		c.498C > G	p.Y166*	P	8.33	1.69
		2		C.611A > G	p.Y204C	LP	8.33	1.69
		2		C.721C > T	p.R241C	P	8.33	1.69
		2		C.728G > A	p.R243Q	P	8.33	1.69
		1		C.208_210delTCT	p.S70del	P	4.17	0.85
		1		C.284_286del	p.I95del	P	4.17	0.85
		1		C.331C > T	p.R111*	P	4.17	0.85
		1		C.353–6T > C	-	P	4.17	0.85
		1		C.464G > A	p.R155H	P	4.17	0.85
		1		C.527G > A	p.R176Q	LP	4.17	0.85
		1		C.907del (p.S303Pfs*38)	p.S303Pfs*38	P	4.17	0.85
Tetrahydrobiopterin deficiency (#233,910, #261,640, #612,716, #264,070, and #261,630)	*PTS* (*612,719)	4	-	-	-	-	16.67	3.39
		1	NM_000,317.3	C.4A > C	p.S2R	P	4.17	0.85
		1		C.259C > T	p.P87S	P	4.17	0.85
		1		C.84–291A > G	-	P	4.17	0.85
		1		C.286G > A	p.D96N	P	4.17	0.85
Short chain acyl CoA dehydrogenase deficiency (#201,470)	*ACADS* (*606,885)	13	-	-	-	-	-	11.02
		3	NM_000,017.4	C.1031A > G	p.E344G	P	23.08	2.54
		2		C.578C > T	p.S193L	VUS	15.38	1.69
		2		C.413delA	p.N138Mfs*36	P	15.38	1.69
		1		C.1130C > T	p.P377L	LP	7.69	0.85
		1		C.795+1G > A	-	LP	7.69	0.85
		1		C.758T > G	p.V253G	US	7.69	0.85
		1		C.172C > T	p.R58*	LP	7.69	0.85
		1		C.286G > A	p.G96S	LP	7.69	0.85
		1		C.220C > T	p.P74S	LP	7.69	0.85
Citrin deficiency (#605,814 and #603,471)	*SLC25A13* (*603,859)	9	-	-	-	-	-	7.63
		4	NM_014,251.2	C.852_855delTATG	p.M285Pfs*2	P	44.44	3.39
		2		IVS16ins3kb	p.A584Vfs*2	P	22.22	1.69
		1		C.1048G > A	p.D350N	VUS	11.11	0.85
		1		C.1638_1660dup23 (p.A554Gfs*17)	p.A554Gfs*17	P	11.11	0.85
		1		C.1750_1751ins3kb	-	P	11.11	0.85
3-methylcrotonyl CoA carboxylase deficiency (#210,200 and #210,210)	*MCCC2* (*609,010)	6	-	-	-	-		5.08
		1	NM_022,132.4	C.1103delG	p.G368Vfs*70	LP	16.67	0.85
		1		C.1550G > A	p.G517E	VUS	16.67	0.85
		1		C.1061C > T	p.T354l	VUS	16.67	0.85
		1		C.1599T > A	p.D533E	VUS	16.67	0.85
		1		C.730C > G	p.P244A	VUS	16.67	0.85
		1		C.1144_1147inv	p.K382_K383delinsF*	P	16.67	0.85
Isovaleric acidemia (#243,500)	*IVD* (*607,036)	5	-	-	-	-	-	4.24
		1	NM_002,225.5	C.158G > C	p.Arg53Pro	P	20.00	0.85
		1		C.349G > A	p.Glu117Lys	VUS	20.00	0.85
		1		c.214G > A	p.D72N	VUS	20.00	0.85
		1		C.631A > G	p.T211A	LP	20.00	0.85
		1		C.865G > A	p.G289R	LP	20.00	0.85
Citrullinemia type I (#215,700)	*ASS1* (*603,470)	4	-	-	-	-	-	3.39
		2	NM_000,050.4	C.1087C > T	p.R363W	P	50.00	1.69
		1		C.748C > T	p.L250F	VUS	25.00	0.85
		1		C.11A > G	p.K4R	VUS	25.00	0.85
2-methylbutryl CoA dehydrogenase deficiency (#611,283)	*ACADSB* (*610,006)	3	NM_001,609.3	C.1165A > G	p.M389V	P	100.00	2.54
Very long chain acyl CoA 377 dehydrogenase deficiency (#609,016)	*ACADVL* (*609,575)	3	-	-	-	-	-	2.54
		1	NM_00,001 8.4	C.621_622+9del	-	P	33.33	0.85
		1		c.622 + 14del	-	VUS	33.33	0.85
		1		c.1531C > T	p.R511W	LP	33.33	0.85
Multiple acyl-CoA dehydrogenase deficiency (#231,680)	*ETFB* (*130,410)	3	-	-	-	-	-	2.54
		1	NM_001,985.2	C.340_342del	p.Lys114del	VUS	33.33	0.85
		1		c.253C > T	p.Arg85Ter	P	33.33	0.85
		1		c.82G > A	p.Gly28Ser	VUS	33.33	0.85
Hypermethioninemia (#250,850)	*MAT1A* (*610,550)	3	-	-	-	-	-	2.54
		1	NM_000,429.3	C.755T > C	p.I252T	LP	33.33	0.85
		1		C.314A > T	p.N105I	VUS	33.33	0.85
		1		C.386A > G	p.D129G	VUS	33.33	0.85
Glutaric acidemia type Ⅰ (#231,670)	*GCDH* (*608,801)	2	-	-	-	-	-	1.69
		1	NM_000,159.3	C.532G > A	p.G178R	P	50.00	0.85
		1		C.1244–2A > C	-	P	50.00	0.85
Propionic acidemia (#606,054)	*PCCA* (*232,000)	2	-	-	-	-	-	1.69
		1	NM_00,028 2.4	C.819+1G > A	-	LP	50.00	0.85
		1		C.1850T > C	p.L617P	VUS	50.00	0.85
Tyrosinemia type Ⅲ (#276,710)	*HPD* (*609,695)	2	-	-	-	-	-	1.69
		1	NM_002,150.2	C.893A > C	p.Q298P	VUS	50.00	0.85
		1		C.217T > C	p.S73P	VUS	50.00	0.85
3-methylglutaconyl CoA hydratase deficiency (#614,739)	*SERAC1* (*614,725)	1	NM_03,286 1.3	C.1364C > G	p.T455S	VUS	100.00	0.85
Nonketotic hyperglycinemia (#605,899)	*GLDC* (*238,300)	1	NM_000,170.2	C.2405C_T	p.A802v	P	100.00	0.85

LP: likely pathogenic; P: pathogenic; VUS: variants of uncertain significance; “/” means no changing.

We found that five types of OAMDs in Changsha, with a incidence rate of 1:100,283.3-MCCD, 2-MBDD, and PA were the most common OAMDs. This is different from other reports in other areas of China, where methylmalonic acidemia was reported as the most common organic acid disorder in Zhejiang and Shandong. The incidence rate of methylmalonic acidemia in China is 1:3,960–1:26,000 (Zhou et al., 2018), but no cases have been reported in the Changsha area. Our screening data showed 4,923 newborns were suspected positive, and 286 of them were related to elevated C3 acylcarnitine and its ratio. After second test and the analyzation of urine by gas chromatography-mass spectrometry, there were still 16 indicated positive, and then suspected patients were further diagnosed by gene mutation detection, but only two cases of PA were confirmed. Methylmalonic acid, methylcitric acid and homocysteine are widely known biomarkers of genetic conditions leading isolated or combined MMA and HCY, or PA. However, elevations of methylmalonic acid, methylcitric acid and homocysteine can also be the result of secondary alterations, such as secondary vitamin B12 deficiency (Pajares et al., 2021). Therefore the second-tier test of methylmalonic acid, methylcitric acid and homocysteine is necessary to differentially diagnose the secondary vitamin B12 deficiency when we screening for MMA and HCY.

The deficiency of 3-methylcrotonyl-CoA carboxylase leads to disruption of the leucine metabolic pathway and accumulation of 3-hydroxyisovalerylcarnitine, 3-hydroxycrotonoylglycine, 3-hydroxyisovaleric acid and other metabolities, the metabolic pathways associated with 3-MCCD and other OAMDs were illustrated in Figure 3. 3-MCCD is classified into type I and type Ⅱ, caused by *MCCC1* (OMIM* 609010) gene and *MCCC2* (OMIM* 609014) gene, respectively. To date, at least 66 *MCCC1* and 83 *MCCC2* mutations have been reported (Yang et al., 2015). But there is no case with *MCCC1* gene mutation in our screening results of 300,849 newborns. The current data show that about 90% of patients with 2-MBDD are asymptomatic (Porta et al., 2019). And the conclusion is corroborated in our findings, the conditions of three cases with 2-MBDD we found were normal during the follow-up. A study reported that the incidence rate of IVA was approximately 1:50,000 (Yoon et al., 2005), and in our study, two patients were found in approximately 300,000 neonates, with an incidence rate of 1:150,425. The least common organic acid disorder was GA-I, with an incidence rate of 1:300,849.

In Australia, Germany, and the United States, the overall incidence rate of FAODs is nearly 1:9,300; however, they are uncommon in Asia (Lindner et al., 2010; Shibata et al., 2018). It was also reported that in China, FAODs are very unusual, accounting for only 13% of the total IMDs (Han et al., 2015). However, research on the incidence of IMDs in the Chinese mainland population indicated that FAODs had a high incidence of 9.16 per 100,000 births (Deng et al., 2021). Our findings were consistent with these results. FAODs have the highest incidence rate of IMDs in Changsha, with an overall incidence rate of 1:9,705. PCD is not only the most common fatty acid oxidation disorder, but also the most common IMD in this study, and its incidence rate even exceeded that of HPA, reaching 1:13,675, which is consistent with the findings from the Quanzhou area (Lin et al., 2019). PCD is an autosomal recessive disorder of FOADs, due to mutations in *SLC22A5* gene (OMIM* 603,377), which encodes the carnitine transporter protein, the biochemical metabolic pathway was shown in Figure 3. Carnitine transporter protein has a high affinity for carnitine in the cell membrane. In our research, c.1400C > G, c.51C > G, and c.760C > T were the hotspot mutation sites of *SLC22A5*, with the mutation frequencies of 24.32, 21.62, and 16.22% (Table 10), respectively, which is consistent with the literature (Guo et al., 2018). 15 kinds of mutations were found and the overall mutation numbers associated with PCD are 37, which accounting for 31.36% in total mutations of IMDs in Changsha (Table 10), that are the most mutations discovered in our research. However, our research is different from Suzhou, which found nine kinds of mutation associated with PCD, and PCD is not the most common disorder in Suzhou either (Wang et al., 2019). SCADD is the second most common fatty acid oxidation disorder, with an incidence rate of 1:42,978. C.1031A > G in *ACADS* (OMIM* 606,885) gene is the most common mutation, accounting for 23.08% in all mutations associated with SCAD found in Changsha. The newborn screening programs in Australia (Wilcken et al., 2009) and Massachusetts (Waisbren et al., 2008) have followed SCADD infants detected on the basis of an elevated C4 acylcarnitine in newborn blood spots and have found that most of the infants grow and develop normally. And in those that do not, clinical disease can often be attributed to another cause. These data, which suggest that SCADD does not cause disease in the great majority of cases. However some researches reveal that short chain fatty acids are very important for maintaining mucosal dynamic balance, their deficiency may affect the pathogenesis of a diverse range of diseases, from allergies and asthma to cancers, autoimmune diseases, metabolic diseases, and neurological diseases (Tan et al., 2014; van der Hee and Wells, 2021). It is important to pay close attention to the level of short chain fatty acids.

In summary, we report the incidence, disease spectrum, and genetic characteristics of neonatal IMDs for the first time in Changsha. FAODs had the highest incidence rate of IMDs in Changsha, which is contrary to previous reports. This may be due to variation in the different regions and populations. The most common IMDs were PCD, HPA and SCADD. Finally, the screening of neonatal IMDs by MS/MS in Changsha is conducive to early diagnosis and intervention. And our findings show newborn screening by MS/MS may miss part of the IMDs, the performance of our newborn screen program needs to be improved, and the combination of MS/MS with gene screening is necessary in future.

## Data Availability

The datasets for this article are not publicly available due to concerns regarding participant/patient anonymity. Requests to access the datasets should be directed to the corresponding author.

## Glossary

**AAMDs**: amino acid metabolic disorders
**ASA**: argininosuccinate aciduria
**BH4D**: tetrahydrobiopterin deficiency
**BKD**: beta-ketothiolase deficiency
**CACTD**: carnitine/acylcarnitine translocase deficiency
**CD**: citrin deficiency
**CIT-I**: citrullinemia type Ⅰ
**CPT-ID**: Carnitine palmitoyltransferase Ⅰ deficiency
**CPT-IID**: Carnitine palmitoyltransferase Ⅱ deficiency
**DLDD**: Dihydrolipoamide dehydrogenase deficiency
**EE**: ethylmalonic encephalopathy
**FAODs**: fatty acid oxidation disorders
**GA-I**: glutaric acidemia type Ⅰ
**H-ARG**: arginemia
HCSD: holocarboxylase synthetase deficiency
**HCY**: homocystinuria
**HHHS**: hyperornithinemia-hyperammonemia-homocitrullinuria syndrome
**HIBCHD**: β-hydroxybutyryl-CoA dehydrogenase deficiency
**H-MET**: hypermethioninemia
**HMGD**: 3-hydroxy-3-methylglutaryl-CoA lyase deficiency
**H-ORN**: hyperornithinemia
**HPA**: hyperphenylalaninemia
**H-PRO**: hyperprolinuria
**H-TYR**: tyrosinemia
**IBDD**: isobutyryl-CoA dehydrogenase deficiency
**IMDs**: inherited metabolic disorders
**IVA**: isovaleric acidemia
**LCHADD**: long chain hydroxyacyl CoA dehydrogenase deficiency
**MADD**: multiple acyl-CoA dehydrogenase deficiency
**MAL**: malonic acidemia
**MCADD**: medium chain acyl CoA dehydrogenase deficiency
**MMA**: methylmalonic acidemia
**MMSDHD**: methylmalonate semialdehyde dehydrogenase deficiency
**MS/MS**: tandem mass spectrometry
**MSUD**: maple syrup urine disease
**NKHG**: nonketotic hyperglycinemia
**OAMDs**: organic acid metabolic disorders
**PA**: propionic acidemia
**PAHD**: Phenylalanine hydroxylase deficiency
**PCD**: primary carnitine deficiency
**SCADD**: short chain acyl CoA dehydrogenase deficiency
**TFP**: trifunctional protein deficiency
**TYR-III**: tyrosinemia type Ⅲ
**UCD**: urea cycle disorder
**VLCADD**: very long chain acyl CoA dehydrogenase deficiency
**2-MBDD**: 2-methylbutryl CoA dehydrogenase deficiency
**3-MCCD**: 3-methylcrotonyl CoA carboxylase deficiency.
**3-MGAD**: 3-methylglutaconyl CoA hydratase deficiency.
